# Mitochondria and Central Nervous System Disorders

**DOI:** 10.3390/biom12101414

**Published:** 2022-10-03

**Authors:** Tomas Knedlik, Marta Giacomello

**Affiliations:** 1 Department of Biology, University of Padua, Via Ugo Bassi 58b, 35100 Padua, Italy; 2 Department of Biomedical Sciences, University of Padua, Via Ugo Bassi 58b, 35100 Padua, Italy

Mitochondria are semi-autonomous, membrane-bound organelles present in the cytoplasm of nearly all eukaryotic cells. Their “most classic” role is to provide the cells with the energy needed to sustain the organism’s life; however, they do not only function as cell “power stations”. Mitochondria regulate complex processes in cell homeostasis—they are a hub for a number of cell signaling cascades and house several metabolic pathways. Not surprisingly, if these processes become dysfunctional, they often impair mitochondria functions and eventually lead to various types of mitochondrial pathological phenotypes.

This Special Issue is focused on the link between mitochondrial dysfunctions and central nervous system (CNS) disorders: the contributions presented here, comprising four original research papers and four review articles, cover various topics, ranging from glutamate toxicity to schizophrenia.

An old proverb says that “excess of everything is bad”. Glutamate excitotoxicity is a common culprit of numerous neurodegenerative diseases. Polster et al. [1] developed a live-cell calpain activity reporter to analyze whether glutamate increases protease activity in neuronal cells prior to delayed calcium deregulation (DCD). First, they suggest that DCD susceptibility might be modulated by thus-far-unknown protease(s) cleaving the reporter probe. Second, their data indicate that excitotoxic glutamate induces the activation of calpain-independent neuronal protease activity prior to the derangement of calcium homeostasis (and mitochondrial bioenergetic function).

Some researchers like proteins, while others prefer lipids; cardiolipin (CL) is a major lipid component of the inner mitochondrial membrane (IMM). CL is also found in the outer mitochondrial membrane (OMM), specifically in the contact sites formed between the OMM and IMM. Similar structures are mitochondria-associated membranes (MAMs), which are in contact with the endoplasmic reticulum (ER). Manganelli et al. [2] demonstrated that CL content in MAMs fraction was significantly increased after autophagy induction. Moreover, using neural SKNB-E-2 cells, they confirmed that CL associates with MAM components during autophagosome formation. As autophagy is altered in some neurodegenerative diseases, these findings may provide new perspectives on the etiology of neurodegenerative diseases and on pharmacological avenues to preserve CL content in the disease.

The opening of mitochondrial permeability transition pores (MPTPs) is a major pathophysiological mechanism of ischemic brain pathology. The modulation of MPTPs may thus represent a potential target for neuroprotection during ischemic brain injuries. Therefore, Skemiene et al. [3] studied whether metformin, phenformin, and other inhibitors of complex I of the mitochondrial electron transfer chain might protect against ischemia-induced cell death in brain slice cultures by suppressing MPTPs. The authors demonstrated that the inhibitors prevented hypoxia-induced necrosis in brain slice cultures and may lead to brain cells’ survival under ischemic conditions.

The last research paper describes “the importance of being balanced”. Schizophrenia is a complex mental disorder defined by continuous or relapsing episodes of psychosis. Bryll et al. [4] investigated the relationship between the level of metabolites in the brain and the clinical status of patients to potentially distinguish between schizophrenia and personality disorders. Their data show that decreased activity of brain metabolites is accompanied by increased peripheral oxidative stress and deterioration of the clinical status of people with schizophrenia. Taking into account these results, they suggest the use of brain metabolite levels as “biomarkers” to improve diagnosis.

“Opportunities neglected can never be recovered” could be the motto of the first review by Gasparotto et al. [5]. Their review deals with the neglected role of nuclear and cytoplasmic players in mitochondria-related nervous system disorders. In the first part of the review, they explore a relationship between mitochondria and chromatin plasticity that enables a cell to promptly respond to (extra)cellular signals without the need to alter its genome. The authors explain that the nuclear changes impacting mitochondria are often associated with an abnormal phenotype linked to altered neurodevelopment. Then, they focus on subcellular trafficking, which regulates several crucial processes, including autophagy and mitophagy. Naturally, the impairment of subcellular trafficking machinery can lead to serious CNS diseases, as they discuss in the second section.

Grespi et al. [6] investigate the interplay of microtubules with mitochondria–ER contact sites (MERCs) in glioblastoma (GB). Glioblastoma is one of the most aggressive types of tumors which originates within the brain. The authors carried out a meta-analysis to compare grade I and grade IV GB patients in order to elucidate whether there MERC–cytoskeleton crosstalk exists and whether GB progression might be linked to altered cytoskeleton–MERC interaction. According to their analysis, the GB samples (grade IV) are characterized by the altered expression of cytoskeletal and MERC-related genes, which both might provide new hints to better understand GB molecular etiopathogenesis and potentially exploit MERC players as novel GB therapeutic targets.

Maresca et al. [7] focus on optic atrophy caused by the death of retinal ganglion cells, which are highly sensitive to mitochondrial dysfunction. The authors provide an overview and speculate on the potential impact of changes in the interplay among bioenergetics, mitochondrial dynamics, mitochondrial cristae morphology, and MERC crosstalk on the pathogenesis of optic nerve neurodegeneration.

In the last review, Vezzani et al. [8] describe the role of mitochondria molecular signaling in neuroinflammation modulation in CNS diseases. Mitochondria are key players in regulating the inflammatory response and are considered to play a prominent role in the development of neurodegenerative disorders. The authors particularly focus on reactive oxygen species production and pattern recognition receptor (PRR) signaling and suggest potential therapeutic approaches which target mitochondrial pathways involved in inflammation.

In summary, in this concise Special Issue, the readers will find articles that propose new perspectives from which to interpret the contribution of mitochondrial defects in CNS pathological contexts.
